# The Relationship Between Visual Function and Performance in Para Swimming

**DOI:** 10.1186/s40798-022-00412-3

**Published:** 2022-02-04

**Authors:** Daniel Fortin-Guichard, H. J. C. Ravensbergen, Kai Krabben, Peter M. Allen, David L. Mann

**Affiliations:** 1 Department of Human Movement Sciences, Amsterdam Movement Sciences and Institute Brain and Behavior Amsterdam, Faculty of Behavioural and Movement Sciences, Vrije Universiteit Amsterdam, Amsterdam, The Netherlands; 2 Vision and Hearing Sciences Research Centre, Anglia Ruskin University, Cambridge, UK

**Keywords:** Paralympic sports, Swimming, Vision impairment, Evidence-based classification, Decision tree analysis

## Abstract

**Background:**

Paralympic swimmers with vision impairment (VI) currently compete in one of the three classes depending on their visual acuity (VA) and/or visual field. However, there is no evidence to suggest that a three-class system is the most legitimate approach for classification in swimming, or that the tests of VA and visual field are the most suitable. An evidence-based approach is required to establish the relationship between visual function and performance in the sport. Therefore, the aim of this study was to establish the relationship between visual function and performance in VI Para swimming. The swimming performance of 45 elite VI swimmers was evaluated during international competitions by measuring the total race time, start time, clean swim velocity, ability to swim in a straight line, turn time, and finish time. Visual function was measured using a test battery that included VA, contrast sensitivity, light sensitivity, depth perception, visual search, and motion perception.

**Results:**

Results revealed that VA was the best predictor of total race time (*r* = 0.40, *p* < 0.01), though the relationship was not linear. Decision tree analysis suggested that only two classes were necessary for legitimate competition in VI swimming, with a single cut-off between 2.6 and 3.5 logMAR. No further significant association remained between visual function and performance in either of the two resulting classes (all |*r*s|< 0.11 and *p*s > 0.54).

**Conclusions:**

Results suggest that legitimate competition in VI swimming requires one class for partially sighted and another for functionally blind athletes.

## Key points

- This empirical study sought to establish the relationship between visual function and performance in elite Para swimming.
- It was found that the current classification system for visually impaired swimmers may not be fit for purpose, with two classes better capturing the relationship between visual function and performance than three.
- It is recommended that no further vision tests should be added in the classification procedure for swimmers with vision impairment.

## Introduction

Classification is vital in sports to ensure fair competition. Classification is the process of grouping athletes together for competition on the basis of characteristics known to impact performance [1]. For example, a heavy-weight wrestler is likely to have an advantage over a light-weight opponent, and therefore, wrestling uses a classification system that places competitors into weight categories. Following this principle, sports use classification systems to reduce the impact of a range of factors on the outcome of competition, for instance to account for an athlete’s gender, age, or maturation status [1].

Classification systems are necessarily sport-specific. Indeed, while being heavier can be advantageous in some sports, it will be disadvantageous in others (e.g., gymnastic). However, the number of factors that can be controlled for in a given sport is limited. Indeed, there are a limited number of event slots in major competitions (e.g., in the Olympic and Paralympic games; [1]). Also, sports that use too many classes encounter logistical challenges when structuring competition. Moreover, by awarding too many medals, those sports can risk devaluing the worth of an individual medal, especially at the highest level. Therefore, only those factors that have the greatest impact on performance are usually controlled for.

In Para sports for people with impairment, classification is required to account for the degree to which an athlete’s impairment impacts their performance in the sport [2]. Para athletes should compete against others with an impairment that has a comparable impact on their sport performance. Moreover, an athlete’s class should be allocated based on the loss of function resulting from their impairment, and that class should not change as a result of training. Classification of impairment in Para sports was originally based on an athlete’s medical diagnosis (e.g., on the location of a spinal cord injury). A problem with this approach was that it does not consider the likelihood that the impact of impairment on performance would differ depending on the sport. Moreover, a medical condition such as a spinal cord lesion can leave some individuals with more functional ability than others [1]. For these reasons, the International Paralympic Committee (IPC) within its *Athlete Classification Code* requires all member sport federations to develop their own evidence-based system of classification designed to be suitable for their sport [3, 4]. An evidence-based system of classification is a system that generates sport classes on the basis of scientific evidence that demonstrates the relationship between impairment and performance in that given sport [1, 5]. Based on those findings, the sport can determine who should be eligible to compete, and what is the fairest manner by which to place athletes into sport classes.

Most sports for athletes with vision impairment (VI) continue to use an outdated classification system that remains the same across almost all sports, and therefore fails to account for the sport-specific relationship between impairment and performance in each sport. The existing system of classification places eligible athletes into one of the three classes that were designed largely on the basis of the World Health Organisation’s definitions of low vision and blindness. Athletes who are functionally blind (generally those with either no or only marginal light perception) are placed in the B1 class, while athletes in the B2 and B3 classes have progressively better visual function[6].^1^ However, change is on the horizon. VI rifle shooting recently became the first VI sport to implement their own sport-specific system of classification. Research in VI rifle shooting demonstrated that only one class was necessary in that sport, because functionally blind athletes could perform just as well as athletes with much less impairment, presumably because in that sport they can effectively rely on the auditory feedback used in the sport to guide the rifle [8–13]. Research has also begun in other VI sports including football [14, 15], judo [16–21], skiing [22, 23], athletics [24], goalball [25], and swimming [7].

### Classification in VI Swimming

Empirical evidence suggests that the existing system of classification for VI swimming may not be fit for purpose. Studies suggest there may be no difference in the performance of athletes in the S12 and S13 classes (i.e., equivalent to B2 and B3; [26–28]). Both groups perform better than the S11 athletes (i.e., equivalent to B1), suggesting that VI does impact performance, but in a nonlinear fashion. In particular, S11 swimmers take more time than S12s and S13s to turn [26], suggesting that specific aspects of a race might be influenced by their poorer visual function.

It might seem as though the existing evidence comparing the three classes should be sufficient to restructure VI swimming into two rather than three classes, but that is far from the case. There are several reasons why research that simply compares the performance of existing sports classes is not sufficient for designing an evidence-based system of classification [1]. First, a comparison of the existing class system relies on the assumption that the measures of visual function used in that system (visual acuity [VA] and visual field) are the most suitable and only measures needed. That, however, is far from established, with a recent Delphi review revealing that experts in VI swimming feel that classification based only on VA and visual field might not fully capture the impact of VI on swimming performance [7]. Those experts noted that other visual functions such as depth perception, light sensitivity, contrast sensitivity, and motion perception should also be considered. For instance, athletes with impaired depth perception might have a disadvantage in their ability to evaluate their distance to the wall, and therefore, their ability to optimally prepare a turn or finish might be impaired. Similarly, impaired contrast sensitivity could impact the ability of swimmers to navigate if they are less able to identify the black line at the bottom of the pool. It remains possible that a subset of athletes may exist in the S12 and S13 classes who are disadvantaged because of an impairment to a visual function that is important for swimming but is not yet assessed during classification. In that case, those athletes might warrant their own separate sport class.

A second concern about studies that compare existing sport classes is that the sport rules can differ between some classes. In swimming, athletes in the S11 class compete with blackened goggles, whereas athletes in the S12 and S13 classes do not. This rule is in place to ensure that all athletes in the S11 class are reduced to no perception of light, enhancing the likelihood that those with remaining vision have no advantage, and minimising the likelihood of athletes intentionally misrepresenting their vision during classification to unfairly enter the S11 class. This rule may though impact the ability to make inferences about the impairment–performance relationship based on existing race data, because it remains possible that athletes with some remaining vision in the S11 class could in fact perform better if they were allowed to swim without occluding goggles.

A third limitation of an approach that compares the performance of existing sport classes is that it is not possible to identify whether an existing class should be separated into multiple sport classes. For instance, it could be that the swimmers with the poorest VA in the S12 class are at a disadvantage and should either join the S11 class or should be placed in their own separate class. These types of decisions can only be made when knowing each athlete’s specific level of visual function rather than just their sport class.

A fourth limitation when comparing sport classes is that, even if access to the measures of visual function is available, those measures may not be sufficiently reliable for research purposes. The aim of athlete evaluation during classification is to determine which sport class an athlete should be allocated to. Accordingly classifiers sometimes do not establish the exact level of VA or visual field if they have already established the class the athlete will be allocated to, especially when VA is worse than 2.6 logMAR and so the classifier knows that the athlete will be in the S11 class irrespective of any further testing [19, 21]. To properly establish the relationship between visual function and performance, a study is necessary that accurately measures different aspects of vision in all athletes.

An examination of the relationship between VI and performance in swimming should focus on those determinants of swimming performance most likely to be impacted by VI. The overall race time is the most common way of measuring performance in swimming, but there are also specific components of the swim time more likely to be impacted than others and therefore might provide a more sensitive measure to changes as a result of impairment [1]. Indeed, establishing the relationship between determinants of performance and overall swim performance is vital, because knowing which determinants of performance are the most impacted by an impairment is a crucial step in conducting evidence-based classification research [29–32]. Based on their Delphi study canvassing the views of experts in VI swimming, Ravensbergen and colleagues [7] proposed a conceptual model that outlined the determinants of performance in a swimming race most likely to be impacted by VI. That model included the ability of a VI swimmer to optimise their performance in each of the start time, the clean swim velocity (with a specific emphasis on the ability to swim in a straight line in the lane), the turn time, and the finish time. For instance, the start and turn times are likely to be affected by an inability to effectively use the full extent of the allowed distance to streamline underwater (i.e., 15 m), with longer underwater distances in particular at the start associated with better race times [26, 27, 33]. Each of these determinants of swim performance could be impacted in their own right by specific aspects of VI.

The aim of this study was to establish the relationship between visual function and performance in VI Para swimming. To do so, we measured the vision and swimming performance of international-level swimmers with VI. We first sought to establish which visual functions best predicted sports performance (addressing Step 4 in Tweedy et al.’s framework for research needs for evidence-based classification; [31, 32]), and then to characterise the optimal number of sport classes necessary to minimise the impact of VI on the outcome of competition (Step 5 in Tweedy et al.’s framework; [31, 32]). Based on the views of the experts in the existing Delphi study [7], we expected that the relationship between visual function and performance would be better explained by the addition of new visual functions (e.g., CS) than when using VA alone. Further, we expected that at least two classes would be necessary to minimise the impact of impairment on the outcome of competition [26–28].

## Methods

### Participants

Seventy-eight (*N* = 78) international-level VI swimmers (46.2% female; *M*_age_ = 21.3, SD = 6.9, range 13–52) participated in this study. However, to allow a comparison of visual function with performance while controlling for training volume and age, we included only those participants (1) who compete in 100 m freestyle swimming, and (2) for whom training volume and age data were available (*n* = 45; 48.9% female; *M*_age_ = 20.8, SD = 6.8, range 13–52). Table 1 describes the participants who met the inclusion criteria according to sports class (S13, S12 or S11). The study was conducted in accordance with the Declaration of Helsinki. All athletes provided written informed consent prior to participation, with the study approved by the local research ethics committee and the International Paralympic Committee. Parental consent was obtained for participants aged under 18 years.

**Table 1:** Characteristics of VI athletes according to sports classes

Variables	Sport class		
	S13 (*n* = 19)	S12 (*n* = 14)	S11 (*n* = 12)
% women	58^a^	29^a^	58^a^
Mean age (SD)	18.3^a^ (4.1)	21.6^a^ (5.5)	23.8^a^ (9.9)
Mean number of lifetime swimming training hours (SD)	5600^a^ (3329)	8274^a^ (5234)	7170^a^ (3911)
% health condition			
Albinism	16	0	0
Anterior eye	11	7	0
Macula	37	29	0
Nystagmus	0	7	0
Optic nerve	5	21	42
Retinal	5	29	42
Retinal + Macula	26	7	8
Whole eye	0	0	8

Same letters (i.e., a) in superscript indicate no difference after Bonferroni correction (*p* < 0.05) when compared to other groups. For the type of VI, frequencies were too small to run meaningful analyses. *SD* standard deviation

### Measures

Measures of personal characteristics, visual function, and swimming performance were collected for each athlete.

#### Personal Characteristics

##### Developmental History Questionnaire

An adapted version of the Developmental History Athlete Questionnaire (DHAQ; [34]) was used to collect personal information about each athlete. This self-administered questionnaire consisted of 32 questions (one dichotomous, 19 short answers, and 12 multiple-choice responses) that collected general information including the athlete’s age, nationality, age at onset of VI, progression of VI over time, other impairments, participation in other sports, and an estimation of their lifetime training volume in swimming (in total hours). Athletes filled out the questionnaire themselves (or with the help of an assistant of their choosing), after which a member of the research team went through the questionnaire with the athlete to confirm the responses. Participants’ gender was deduced from the competition in which they took part.

#### Tests of Visual Function

The objective of the tests of visual function was to assess each athlete’s habitual level of visual function during competition. The athletes were therefore asked to wear any visual correction (i.e., prescription goggles or contact lenses) that they used during competition. For the same reason, tests of visual function were conducted binocularly rather than monocularly as recommended by the IPC/IBSA position stand [6]. All tests took place in a room with standard room illumination (≈ 200 lx).

##### Visual Acuity (VA)

The Berkeley Rudimentary Vision Test (BRVT; [35]) was used to assess each athlete’s VA in logMAR units. The BRVT is designed to assess VA in individuals with low vision using three types of cards (single tumbling Es, grating, and black/white discrimination) that measure VA by establishing the distance at which the object on the cards can be resolved. The four cards with a single letter E (either 25, 40, 63, or 100 M size) can be presented in one of the four orientations (left, right, up, and down) at different distances to test VA up to 2.6 logMAR. The grating cards contain a series of black and white parallel lines (either 50, 80, 125, or 200 M size) that can be presented in one of the two directions (horizontal or vertical) to measure VA at different distances up to 2.9 logMAR. The black/white discrimination cards are split into black and white sections or are entirely black or white. The task for participants during the BRVT is to verbalise the direction of the E, grating, or location of the black/white areas, respectively. Gratings were only shown when VA was worse than 2.6 logMAR, and black–white discrimination when VA was worse than 2.9 logMAR. When participants were unable to discriminate black from white, the experimenter assessed whether they could perceive light. A pen torch was directed towards their eyes and the athlete was asked to respond whether the light was on or off. ‘Light perception’ was recorded when the athlete responded correctly 3 out of 4 times. Black/white discrimination was nominally defined as 3.5 logMAR, light perception as 3.7 logMAR, and no light perception as 4.0 logMAR [10, 23, 36]. In alignment with the IPC’s VI classification decision making rules for the single-letter E cards, when multiple cards were used, the card yielding the second-best VA score was taken as the athletes’ true VA (to minimise the chance of erroneous scores with a single better-than-expected result). A lower logMAR value indicates better VA.

##### Contrast Sensitivity (CS)

CS was assessed using the Mars number test (Mars Perceptrix, Chappaqua, NY). The Mars number test consists of three charts, each having a sequence of eight rows of six numbers, starting with the highest contrast of 1.92 logCS on the top left and each number successively decreasing in contrast by 0.04 logCS units. The charts were placed almost vertically on a reading stand, with the athlete asked to read out the numbers. The examiner stopped the test when two consecutive incorrect answers were given. The CS threshold was defined as the contrast level of the final correct number minus 0.04 logCS units for each incorrect response prior to the final correct answer. A higher logCS value on the Mars chart indicates better CS. The acuity demands of the Mars chart meant that not all athletes were able to perform the test (*n* = 16). A dummy value of 0.00 logCS was attributed in those cases.

##### Light Sensitivity (LS)

LS was measured as the difference in logCS (using the Mars test) when viewed with standard lighting versus when viewing through a bright light simulated using the Brightness Acuity Tester (BAT) at its brightest setting of 400 foot-lamberts (Marco Ophthalmic, Inc., Jacksonville, FL). The BAT is a hand-held instrument consisting of an internally illuminated small white bowl that the participant holds over one eye. The bowl has a central opening of 12 mm for the participant to look through. The Mars test was performed monocularly on the athlete’s better eye because the BAT only allows monocular testing. All athletes first performed the test under standard lighting conditions. The test was then repeated while looking through the BAT with the light source switched off to assess whether the central opening affected test performance. Finally, the light source was turned on and the Mars test repeated. The difference in logCS between normal lighting (through the central opening) and bright light was calculated. Results were transformed logarithmically because the distribution was skewed towards zero. A bigger logarithmic difference indicates higher LS. A dummy value of zero was allocated to athletes who were not able to perform the test (*n* = 16; i.e., highest possible value on the test), largely because their visual function/CS was so bad that bright lighting made little difference to their ability to see.

##### Depth Perception (DP)

A modified version of a Howard–Dolman test was specifically created for individuals with low vision to assess DP [37]. One stationary white rod (20 mm diameter) was placed 300 mm to the left of an identical target placed on a rail (both reaching 555 mm above the table surface). The athlete could move the sliding target with a pole attached to the slider. Athletes were seated 1.5 m away from the stationary target. The background of the test was black, and a black barrier blocked the lower part of the athlete’s view to remove any visual cues from the base of the targets and the sliding rail. The sliding target was placed at the far end of the slider (approximately 400 mm further away than the stationary target). The athlete was instructed to move the sliding target until it was the same distance from them as the stationary target. This task was repeated twice more, and the distance between the centres of the two targets was determined in millimetres. The sliding target was then moved to the end of the slider closest to the athlete (approximately 400 mm closer than the stationary target). Again, the athlete was instructed to move the target until it was equidistant with the stationary target. This task was repeated twice more, with the distance between targets determined. The mean absolute value across all six trials was used as the dependent variable. Results were transformed logarithmically because the distribution was skewed towards zero. A lower logarithmic value indicates better DP. A dummy value equal to the maximum observed mean distance plus 10% was allocated to athletes not able to perform the test (*n* = 17).

##### Visual Search (VS)

A test of VS was developed in Psykinematix to assess the ability of participants to search for a target (i.e., whether a circle was present in a grid of squares using Sloan-style characters; [38, 39]). The test was conducted on a 27″ Apple display screen with a refresh rate of 60 Hz and a resolution of 2560 × 1600 pixels. The task was separated into three difficulty levels, with six trials per level. Athletes always started with the easiest level and only continued to the next level if they answered four out of six trials correctly. For the first level, a 3 × 3 grid was shown (subtending 18.5° of visual angle) with black shapes (each subtending 8.3°, equivalent to 2.0 logMAR) on a white background. At the intermediate level, an 8 × 8 grid was used with shapes subtending 2.6° (equivalent to 1.5 logMAR). The most difficult level consisted of a 15 × 15 grid with shapes subtending 0.83° (equivalent to 1.0 logMAR). Each trial was presented for a maximum of 30 s, during which the athlete was required to respond as quickly as possible using the up or down key on a keyboard to, respectively, indicate whether a circle was present or absent. The circle was present in two-thirds of trials. The order of the present and absent trials was randomly selected by Psykinematix, as was the location of the circle. The response time for the most difficult level completed by the athlete (considering only trials where the target was present) was used for analyses, as it was the only measure not correlated with VA, providing a potentially unique contribution to the analysis (i.e., response time in other levels and response accuracy in all levels correlated significantly with VA). Results were transformed logarithmically because the distribution was skewed towards zero. A lower logarithmic value indicates better VS. A dummy value equal to the maximum recorded score plus 10% was allocated to athletes not able to perform the test (*n* = 15).

##### Motion Perception (MP)

A test of global motion coherence was designed in Psykinematix (KyberVision Japan LLC) specifically for individuals with VI and conducted on the same display monitor as the VS test [21, 40]. One hundred dots subtending 1.66° of visual angle were presented in a square envelope of 25°. The lifetime of each dot was 200 ms and the movement speed was 6°/s. Dots moved either vertically (up or down) or in any other random direction, with the percentage of dots moving in a coherent direction (up or down depending on the trial) systematically manipulated to find the threshold proportion of dots that needed to be coherent for the athlete to correctly identify the global direction in which the dots were moving. Athletes were asked to determine the general direction of the movement of the dots from two options (upward or downward motion) using the upward and downward key on the keyboard. Each trial was presented for a maximum of 8 s, within which time athletes were required to respond.

The test started with a set of six familiarisation trials where all 100 dots were moving in the same direction (i.e., 100% coherence). When athletes provided at least four correct responses, the full test protocol commenced. A 1-up-2-down staircase procedure with five reversals was used, where the coherence levels of the final four reversals were averaged to determine the threshold coherence level where global motion could be detected in 66.7% of presentations. Within the staircase, global motion coherence started at 100% coherence and decreased by 25% prior to the first reversal, and decreased or increased by 10% after the first reversal. The test was aborted if six successive incorrect responses were provided at 100% coherence.

Initial inspection of the results showed a dichotomous pattern, with athletes recording motion coherence levels either similar to or below that of a control group of unimpaired individuals tested previously. Accordingly, the results were dichotomised as ‘normal’ or ‘impaired’. A cut-off was established between the two categories at 56% threshold coherence using k-means cluster analysis, with a higher threshold reflecting poorer MP. Participants not able to perform the test were classified as ‘impaired’ (*n* = 11).

#### Performance Measures

For measures of swimming performance, the objective was to assess aspects of the athletes’ performance that experts had nominated were likely to be impacted by VI during competition [7]. In-competition data were collected at international swim meets between June 2016 and April 2017. Competition data were only included if collected within the 6 months before or after we tested that athlete’s visual function. The performance measures were: (1) best race time; (2) start time; (3) clean swim velocity; (4) turn time; (5) finish time; and (6) mean lateral position in the lane. Note that athletes in the S11 sports class compete with blackened swimming goggles and so there results were recorded without any vision (i.e., it remains possible that the performance of these athletes *could* be better than what was measured if they were to swim without blackened goggles).

The *best race time* was defined as each athlete’s fastest 100 m freestyle race time recorded at an international competition within 6 months of when we tested their vision. Data were obtained from official race results held by World Para Swimming, the International Federation for Para swimming. The best race time for each athlete was standardised according to the Olympic world record for that athlete’s gender as follows (with the world record representing a score of 100%):

$$\text{Standardised performance} \, =\,\frac{\text{Best race time}}{\text{World record time}}\text{*100\%}$$

To assess other aspects of swimming performance, video footage of the swimmers was recorded using GoPro 3 cameras during 100 m freestyle races at international competitions in 50 m pools (side-on cameras unless stated otherwise; when multiple races were available for a participant, the fastest time for each race segment was used). *Start time* was defined as the time taken from the start of the race to that to reach the 15 m flags. *Clean swim velocity* was defined as the average speed (m/s) across the 15th to 45th meter and the 55th to 95th meter markers. *Turn time* was the time taken to travel from the 45th to the 55th meter marks (with the turn at the 50 m mark). *Finish time* was the time taken to swim through the final five meters. Finally, *mean lateral position* was the average absolute distance of the swimmer from the centre of the lane (in cm). Video footage was recorded from an elevated position at the end of the pool so that the lateral position of the swimmer in the lane could be manually digitised throughout the race (1 Hz, Kinovea, Bordeaux, France; https://www.kinovea.org/). Note that footage from nine participants were not clear enough to produce usable data on at least one of those measures. Data were not replaced in those cases. The performance measures showed excellent inter- (ICC = 0.92–0.96) and intra-rater reliability (ICC = 0.88–0.95) when tested on 20% of our dataset (excluding best race time given that it was extracted from official race results).

### Procedure

Each athlete was tested individually on the tests of visual function. Athletes were either tested at competition between training and races, or outside of competition. Athletes were free to choose their preferred test time. VA was always tested first, and light sensitivity last, but the order of the other tests was not necessarily controlled. Testing of visual function lasted approximately one hour, but could be shorter for athletes with rudimentary vision who were not able to perform most tests. Swimming performance was determined from official race results and from video footage after all athletes had completed their testing of visual function.

### Data Analysis

Analyses were conducted using R Studio Version 1.3 (RStudio, PBC, Boston, MA; https://rstudio.com/products/rstudio/), supported by R version 4.0.0 (The R Foundation, Vienna, Austria; https://www.r-project.org/foundation/). One-way ANOVAs (with Bonferroni post hoc tests) and Pearson’s Chi-square tests were run on the descriptive statistics to verify sport class homogeneity (Table 1). Correlations were first run to assess the relationship between swimming performance and (1) training volume in hours and (2) age, because these variables could confound the relationship between vision and swimming performance. When significant correlations were present, we ran hierarchical linear regressions to verify whether training volume or age, when forced at step 1, best predicted each performance measure by itself, or if gender added to the prediction when inserted at step 2. Then, when gender added to the quality of the prediction, performance measures were also adjusted for gender by extracting the residuals of those regressions separately by gender (i.e., the performance measures that we report are the residuals of the regression of the confounders on performance). Note that the supplementary steps regarding the adjustments for gender were not done for *Best race time* because gender was already taken into account in the standardisation procedure by using the gender-specific world record.

#### Relationship Between Visual Function and Performance for All Athletes

Correlations were conducted to assess the relationship between measures of visual function and swimming performance (point biserial correlation in the case of MP). Where appropriate, partial correlations were conducted to control for other measures of visual function to establish the *unique* contribution of each vision measure on performance. For each performance measure related to visual function after partial correlation, an identical series of three analyses was carried out. First, a decision tree algorithm using the *ctree* function from the *partykit version 1.2-7* R package was run to find if any measure of performance could be split according to the appropriate measure of visual function. The number of splits and the border between splits are reported, as well as Welch t-tests (i.e., correction for unequal variances) to compare swimming performance above and below the split. Second, when the decision tree found at least one split in performance, bootstrapping of the decision tree with replacement 10,000 times was run to confirm the validity of the split [21]. The distribution of the splits from the 10,000 trees is reported. Third, performance was dichotomised (*low* or *high* performance) according to optimal classification. Dichotomisation was done using the groups created based on the decision trees and bootstrapping. Using those two groups, optimal classification of those ‘high performing’ and ‘low performing’ athletes was determined at Youden’s J (i.e., indicating optimal sensitivity and specificity). This binary performance outcome was included in a hierarchical logistic regression to determine whether the incorporation of additional measures of visual function would improve the classification of swimmers as those with low or high performance as opposed to what was possible with a single measure of visual function. For all analyses, the alpha threshold was fixed at 0.05.

## Results

### Confounding Factors that Could Influence Swimming Performance

Table 2 presents the correlations between the measures of swimming performance and those variables that might confound any relationship between visual function and performance (i.e., age and training volume). Results revealed that training volume correlated significantly with five of the six measures of performance (all except for the mean lateral position in the lane). The age of the swimmer correlated significantly with three of the six performance measures, though for five of the six performance measures, the strength of the correlation was weaker than it was between training volume and performance (meal lateral position being the exception). Note that age and training volume were highly correlated with each other (*r* = 0.79, *p* < 0.001).

**Table 2:** Relationship between potential confounders and performance measures

Confounders	Performance measures					
	BRT (*n* = 45)	ST (*n* = 40)	CSp (*n* = 40)	TT (*n* = 42)	FT (*n* = 41)	MLP (*n* = 38)
Training volume (hours)	− .42**	− .50**	.34*	− .52***	− .34*	.01
Age	− .31*	− .38*	.28	− .38*	− .17	.14

*BRT* best race time, *CSp* clean speed, *FT* finish time, *MLP* mean lateral position, *ST* start time, *TT* turn time

*p < 0.05; **p < 0.01; *** *p* < 0.001

Because training volume had the highest correlation of the two confounders in almost all cases, we chose to first control for training volume. The best model fit for each performance measure was a quadratic fit so we adjusted using that. To check whether age should also be controlled for, partial correlations were run between age and each performance measure while controlling for training volume. Results indicated no remaining associations (|*r*s|≤ 0.27, *p*s ≥ 0.14). This suggested that performance need only to be adjusted according to the athlete’s total training volume in hours. Next, hierarchical regressions confirmed that gender significantly improved prediction of performance in all cases except for mean lateral position in the lane (Table 3). As a result, all those performance measures reported forthwith are adjusted for both training volume and gender by reporting the standardised residuals of the regression of training volume on each performance measure. The residuals can be interpreted as follows: zero represents the level of performance expected based on the swimmer's training volume; a positive value represents poorer performance than what would be expected based on their training volume (with + 1 corresponding to a race time one standard deviation slower than expected); and a negative value represents better performance than expected based on their training volume (i.e., faster race time). For mean lateral position, a lower value represents a swim closer to the centre of the lane.

**Table 3:** Improvement of the prediction of the hierarchical linear regressions of training volume and gender on all performance measures, with training volume being forced into the model at step 1, and gender at step 2

Confounders	Performance measures (Adjusted *R*^2^)				
	ST (*n* = 40)	CSp (*n* = 40)	TT (*n* = 42)	FT (*n* = 41)	MLP (*n* = 38)
Step 1 (Training volume)	.21**	.12	.27**	.08	.01
Step 2 (Gender)	.50***	.52***	.49***	.26**	.01

*CSp* clean speed, *FT* finish time, *MLP* mean lateral position, *ST* start time, *TT* turn time

Significant effect at step 1 indicates an *R*^2^ different from 0 with training volume only, and a significant effect at step 2 indicates an improvement from step 1 to step 2 in the quality of prediction. Note that best race time was not included in this set of analysis as it was already adjusted to gender by the world record standardisation procedure

*p < 0.05; **p < 0.01; *** *p* < 0.001***

### Relationships Between Visual Function and Swimming Performance

#### Excluding Missing Values for Measures of Visual Function

Correlation analyses presented in Table 4 reveal VA to be significantly associated with four of the six performance measures. Contrast sensitivity was the only other visual measure related to performance, with significant associations with the finish time and mean lateral position in the lane. Some measures of visual function were correlated with each other, with VA and CS showing the strongest association (*r* = − 0.84, *p* < 0.001; see Table 5). Partial correlations between CS and performance measures while controlling for VA confirm that there were no remaining associations for any of the performance measures (|*r*s|≤ 0.14, *p*s ≥ 0.411). These results provide the first suggestion that VA remains the best candidate measure of visual function for predicting swimming performance.

**Table 4:** Correlation between visual functions and standardised residual performance measures with and without missing values

Visual functions	Performance measures					
	BRT	ST	CSp	TT	FT	MLP
Excluding missing values						
Visual acuity	.40**	.31*	− .23	.26	.50***	.71***
Contrast sensitivity	− .30	− .27	.20	− .15	− .49**	− .60***
Light sensitivity	− .06	.08	.15	− .07	− .01	− .21
Depth perception	.06	− .18	.16	− .13	− .11	− .10
Visual search	− .20	.15	.33	− .30	− .05	− .30
Motion perception	.09	− .26	− .32	.02	.21	.37
Including missing values (using dummy values)						
Visual acuity	.40**	.31*	− .23	.26	.50***	.71***
Contrast sensitivity	− .27	− .24	.20	− .15	− .43**	− .54***
Light sensitivity	.27	.32*	− .19	.19	.31*	.41**
Depth perception	.32*	.19	− .17	.15	.41**	.47**
Visual search	.35*	.27*	− .18	.18	.56***	.53***
Motion perception	− .06	− .23	− .11	− .06	− .07	− .15

All coefficients are Pearson correlations. All performance measures except for mean lateral position in the lane are adjusted according to training volume and gender. The number of participants included in each correlation vary between *n* = 24 and *n* = 45

*BRT* best race time, *CSp* clean speed, *FT* finish time, *MLP* mean lateral position, *ST* start time, *TT* turn time

*p < 0.05; **p < 0.01; ***p < 0.001

**Table 5:** Correlation matrix of visual functions for all participants with and without missing values

Visual functions	1	2	3	4	5
Excluding missing values					
1. Visual acuity					
2. Contrast sensitivity	− .84***				
3. Light sensitivity	− .03	− .44*			
4. Depth perception	.44*	− .38	− .10		
5. Visual search	.25	.04	.10	.02	
6. Motion perception	− .38	.10	− .04	− .23	− .46*
Including missing values (using dummy values)					
1. Visual acuity					
2. Contrast sensitivity	− .79***				
3. Light sensitivity	.59***	− .76***			
4. Depth perception	.80***	− .80***	.55***		
5. Visual search	.77***	− .62***	.49***	.64***	
6. Motion perception	− .38**	.38**	− .26	− .51***	− .50**

The number of participants included in each correlation vary between *n* = 27 and *n* = 45

*p < 0.05; **p < 0.01; ***p < 0.001

#### Including Missing Values (Using Dummy Values) for Measures of Visual Function

We ran additional correlations when allocating dummy values to participants who were not able to complete each test of visual function. All the significant correlations found previously remained (i.e., between VA, CS, and performance), in addition to correlations between LS, DP, VD, and measures of swimming performance (see Table 4). However, almost all measures of visual function significantly correlated with each other when dummy values were allocated (Table 5). Partial correlations were conducted to determine whether any of the measures of visual function remained correlated with swimming performance while controlling for VA. Results revealed that only an association between DP and mean lateral position in the lane remained when controlling for VA (*r* = − 0.33, *p* = 0.049; all other associations between visual function and performance, |*r*s|≤ 0.29, *p*s ≥ 0.07). These results provide further support for VA being the best predictor of swimming performance, but also suggest a potential association with DP.

#### Measures of Performance

VA was used as the main measure of visual function for further analyses given its primacy as the key predictor of performance. Partial correlations were conducted between VA and each of the performance measures while controlling for the best race time (i.e., theoretically and practically the most relevant measure of performance in swimming). Results revealed no further association between VA and start time (*r* = 0.05, *p* = 0.75), clean speed (*r* = 0.20, *p* = 0.23), or turn time (*r* = 0.01, *p* = 0.95). Those measures of performance were therefore dropped from further analyses. However, VA remained related to finish time (*r* = 0.35, *p* = 0.026) and mean lateral position in the lane (*r* = 0.68, *p* < 0.001). In the following subsections, we explore how visual functions, with a specific emphasis on VA, are related to each of the three remaining measures of performance. Analyses are fully described for the best race time, but for the sake of brevity, only a summary of the findings (using the same analyses) are presented for finish time and mean lateral position.

##### Best Race Time

**Fig. 1:**
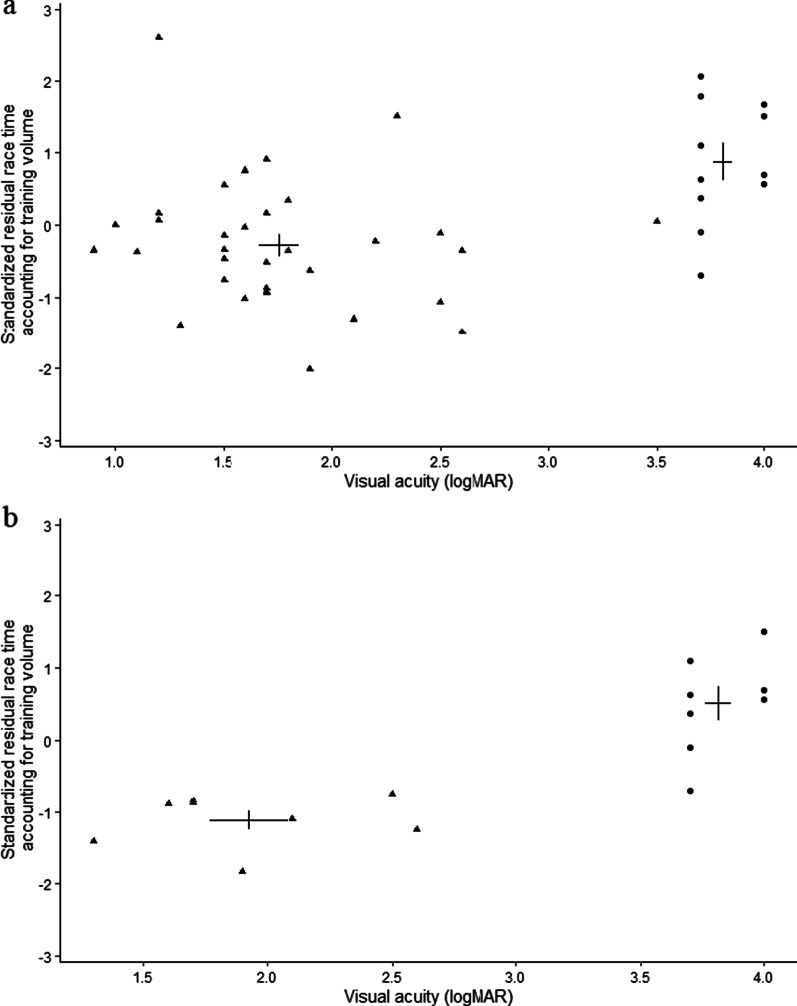
Residual race time and VA for **a** all participants, and **b** the eight best performers in each group created on the basis of the decision tree analysis. Circles represent participants with VA > 3.5 logMAR, and triangles represent participants with VA ≤ 3.5 logMAR. The crosses represent the means of each group, with the horizontal and vertical branches representing the standard errors of the means of VA and residual race time, respectively

The decision tree analysis revealed that a single split at a VA of 3.5 logMAR provided the best possible split in the race times of the swimmers (Fig. 1a; note though the lack of data between 2.6–3.5 logMAR).^2^ Performance was significantly poorer in the group with VA worse than 3.5 logMAR (*n* = 11; *M* = 0.872) than it was in the group with VA better than or equal to 3.5 logMAR (*n* = 34; *M* = − 0.281), *t*(17.64) = 3.88, *p* < 0.002, *d* = 1.35. The algorithm found no further split based on VA. Because swimming races typically contain eight competitors, Fig. 1b illustrates the top eight performers from each group. Results show that even the best athlete from the > 3.5 logMAR group would not have made the final if conducted for the top-8 performers in the ≤ 3.5 logMAR group.

**Fig. 2:**
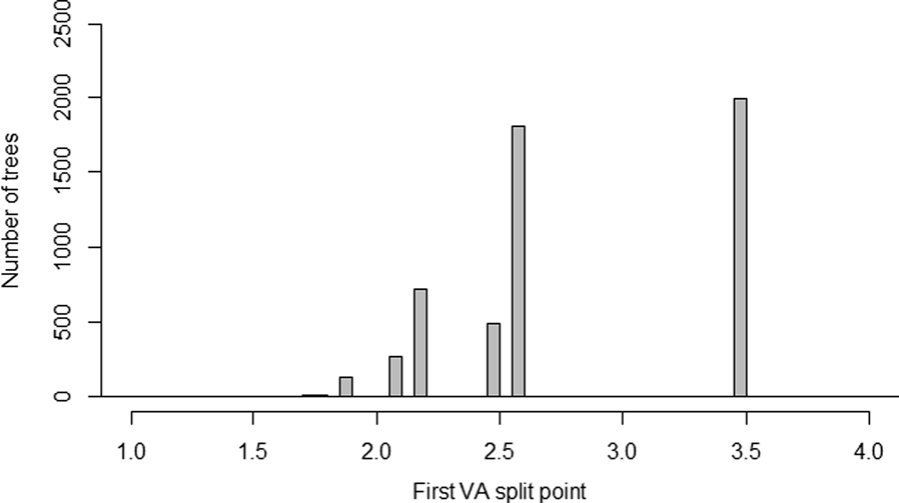
Histogram of the first VA split points of best race time using 10,000 bootstrapped samples. The data split at least once in 5,498 cases

Bootstrapping of the decision tree mostly supported the validity of a single split at 3.5 logMAR. The dataset was found to split at least once in 55.0% of the 10,000 bootstrap samples, with a single split being the most likely outcome (54.3% of all cases). Two splits were found in only 0.7% of cases. Of the trees that found at least one cut-off point (Fig. 2), the majority of the first splits were either at 3.5 logMAR (36.6%) or 2.6 logMAR (33.3%). The next most frequent were 2.2 logMAR (14.2%) and 2.5 logMAR (9.2%).

Having classified the participants into two groups on the basis of VA, we sought to establish whether classification would improve if additional measures of visual function were included. To do so, first the performance of each swimmer was classified as ‘high performing’ or ‘low performing’. The threshold race time for classification was determined by choosing the standardised residual best race time that optimally classified those with VA ≤ 3.5 logMAR as ‘high performing’ and those with VA > 3.5 logMAR as ‘low performing’. Optimal classification occurred at Youden’s J when the standardised residual best race time was 0.352 (sensitivity = 0.85, specificity = 0.82). Performance was indeed poorer in those placed in the low performance group (*n* = 14; *M* = 1.19) than it was in those placed in the high performance group (*n* = 31; *M* = − 0.54), *t*(21.43) = 8.42, *p* < 0.001, *d* = 2.98.

Second, a hierarchical logistic regression revealed that the addition of other measures of visual function did not improve the rate of classification (see Table 6). VA was forced into the regression model at Step 1, and the additional measures of visual function at Step 2. Not surprisingly, VA significantly predicted group membership at Step 1 (*B* = − 1.26, S.E. = 0.39), odds ratio = 0.28 (95% CI = 0.13–0.61), where poorer VA indicated higher odds of being categorised in the low performance group, Nagelkerke *R*^2^ = 0.36 (82.2% of correct classification). Vitally, none of the additional measures added to the quality of prediction at Step 2 (i.e., no further significant predictors and therefore no change in Nagelkerke *R*^2^ nor percentage of correct classification, see Table 6 for regression statistics). Results suggest that the use of VA alone provided the most parsimonious means of separating the group into two classes.^3^

**Table 6:** Hierarchical logistic regression of all visual functions on best race time, with VA being forced into the model at step 1, and other visual functions entered with a stepwise method at step 2

Variables	χ^2^_w_	*p*
Step 1		
**Intercept**	**13.28**	**< 0.001**
**Visual acuity**	**10.56**	**0.001**
Step 2		
**Intercept**	**13.28**	**< 0.001**
**Visual acuity**	**10.56**	**0.001**
Contrast sensitivity	1.61	0.205
Light sensitivity	0.03	0.855
Depth perception	0.82	0.365
Visual search	0.26	0.613
Motion perception	1.68	0.195

Bold indicate predictors kept in the model. *χ*_w_^2^ refers to Wald Chi-Square. Dummy values for CS, LS, DP, VS and MP were used for participants not able to complete those tests. The goodness of fit Hosmer–Lemshow test was not significant, *χ*^2^(7) = 6.67, *p* = 0.464, indicating good reliability of the model

##### Finish Time and Mean Lateral Position

Table 7 presents the main results for finish time and mean lateral position, including the respective decision trees, bootstrapping, and logistic regressions. Three main conclusions differ between best race time and results regarding finish time and mean lateral position. First, the decision tree split finish time and mean lateral position at 2.6 logMAR as opposed to the 3.5 logMAR found for the best race time. Second, the logistic regression to predict high/low performance suggests that VS, LS, DP, and MP can improve the quality of the prediction of finish time above what is possible with VA alone, and can even replace VA at Step 2. However, the increase in percentage of correct classification is marginal (from 85.4 to 87.8%; Table 7). Similarly, VA alone is also not sufficient to predict mean lateral position, with DP contributing to the quality of the prediction (note though the decrease in the quality of correct classification from 89.5 to 86.9%; Table 7). Overall, results from finish time and mean lateral position suggest that other measures of visual function may be related to performance in some aspects of the swimming race, but that their addition does not practically improve the percentage of correct classification into high or low performing athletes.

**Table 7:** Results summary for finish time and mean lateral position

Performance measures	Main outcomes						
	Decision tree split	Performance difference	Trees with at least one split (%)	Frequency of first split	Splitting performance	Logistic regression included at step 1	Logistic regression included at step 2
Finish time	Single split at 2.6 logMAR	> 2.6 logMAR *n* = 12 *M* = 0.939 ≤ 2.6 logMAR *n* = 29 *M* = − 0.364 *t*(26.83) = 5.07, *p* < 0.001, *d* = 1.6	83.92	2.6 logMAR 41.6% 2.1 logMAR 24.4% 2.5 logMAR 10.7% 1.9 logMAR 9.3% Others 14.0%	Youden’s J 0.398 Sensitivity 0.86 Specificity 0.83	Intercept *p* < 0.001 Visual acuity *p* < 0.002 χ^2^ = 15.10 Nag. *R*^2^ = 0.43 % Cor. Class. = 85.4	Intercept *p* = 0.079 Visual acuity *p* = 0.283 Visual search *p* = 0.102 Motion perception *p* = 0.060 Light sensitivity *p* = 0.095 Depth perception *p* = 0.141 χ^2^ = 30.03* Nag. *R*^2^ = 0.72 % Cor. Class. = 87.8
Mean lateral position	Single split at 2.6 logMAR	> 2.6 logMAR *n* = 12 *M* = 0.689 ≤ 2.6 logMAR *n* = 26 *M* = 0.277 *t*(23.79) = 7.37, *p* < 0.001, *d* = 2.5	99.97	2.6 logMAR 44.6% 2.5 logMAR 27.4% 2.2 logMAR 17.8% Others 10.2%	Youden’s J 0.505 Sensitivity 0.85 Specificity 0.91	Intercept *p* < 0.001 Visual acuity *p* < 0.001 χ^2^ = 22.59 Nag. *R*^2^ = 0.61 % Cor. Class. = 89.5	Intercept *p* = 0.997 Visual acuity *p* = 0.009 Depth perception ^a^ *p* = 0.070 χ^2^ = 27.70* Nag. *R*^2^ = 0.71 % Cor. Class. = 86.8

% Corr. Class, percentages of correct classification, Nag. *R*^2^, Nagelkerke *R*^2^

*Indicates a significant change in *χ*^2^ from step 1 to step 2

^a^Predictor is kept in the model despite *p* > 0.05, because the selection criteria was based on the AIC

### Relationship Between Visual Function and Swimming Performance for Athletes with VA ≤ 3.5 logMAR

Further analyses conducted on the subgroup of athletes with VA ≤ 3.5 logMAR created by the original decision tree suggest that further splits are not necessary on the basis of the other measures of visual function.

#### Best Race Time

**Fig. 3:**
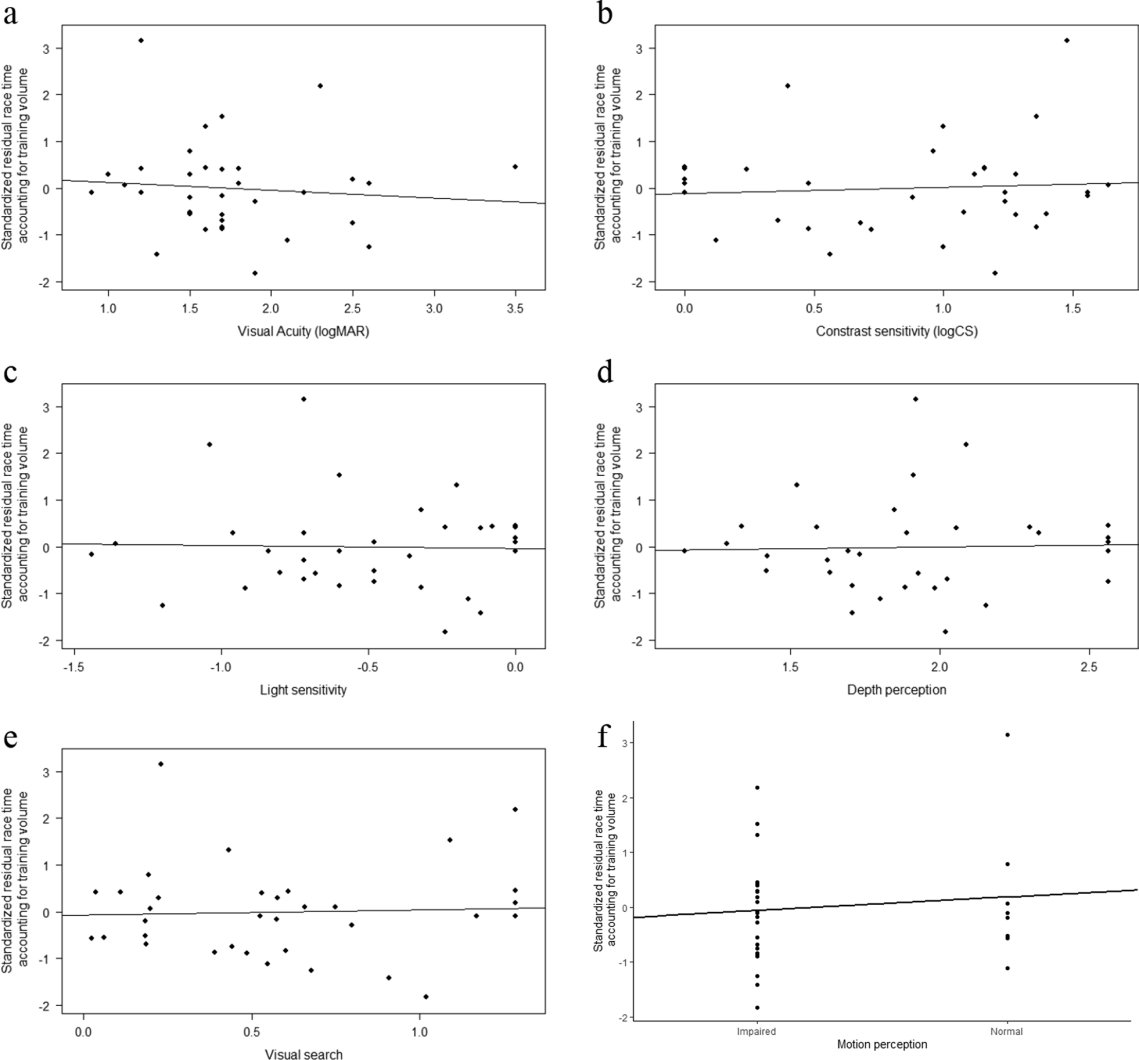
Relationships between residual race time and each of the six measures of visual functions (**a–f**) for athletes with VA ≤ 3.5 logMAR

Correlation analysis considering only athletes with VA ≤ 3.5 logMAR revealed no significant relationship between performance and any of the measures of visual function (|*r*s|< 0.11, *p*s > 0.54; see Table 8 and Fig. 3). This finding suggests that no further split may be necessary on the basis of the other measures of visual function. Note that missing values were replaced with dummy values for CS, LS, DP, VS, and MP.

**Table 8:** Correlation matrix for measures of visual functions and best race time for participants with VA ≤ 3.5 logMAR

Variables	1	2	3	4	5	6
1. Best race time						
2. Visual acuity	− .09					
3. Contrast sensitivity	.07	− .65***				
4. Light sensitivity	− .03	.30	− .64***			
5. Depth perception	.03	.68***	− .68***	.32		
6. Visual search	.05	.53**	− .35*	.20	.39*	
7. Motion perception	.11	− .34	.29	− .14	− .45**	− .45**

* *p* < 0.05; **p < 0.01; *** *p* < 0.001

Having ruled out the need to split the group using *individual* measures of visual function, we used linear multiple regression (enter method) to verify whether a combination of two or more measures of visual function would better predict performance (i.e., when taking into account the influence of other predictors on performance). Results revealed that, taken together, there was no combination of visual functions that was able to significantly predict the best race time, *F*(6, 27) = 0.36, *p* = 0.897, Adjusted *R*^2^ = − 0.13, suggesting that no further split in this subgroup was necessary.

#### Finish Time and Mean Lateral Position

Table 9 summarises the results for finish time and mean lateral position, incorporating the respective correlations and multiple linear regression among athletes with VA ≤ 3.5 logMAR. Note that even if the initial decision tree for mean lateral position and finish time split the data at 2.6 logMAR, the analyses in this section were run on participants with VA ≤ 3.5 to facilitate comparison. The conclusions related to finish time and mean lateral position are the same as those from best race time, suggesting no further split was necessary.

**Table 9:** Results summary for finish time and mean lateral position for participants with VA ≤ 3.5 logMAR

Performance measures	Main outcomes	
	Correlations with visual function measures	Multiple linear regression
Finish time	|*r*s|< 0.15 *p*s > 0.414	*F*(6, 23) = 0.49 *p* = 0.81 Adjusted *R*^2^ = − 0.12
Mean lateral position	|*r*s|< 0.19 *p*s > 0.341	*F*(6, 20) = 0.59 *p* = 0.74 Adjusted *R*^2^ = − 0.11

### Relationship Between Visual Function and Swimming Performance When VA > 3.5 logMAR

When VA was > 3.5 logMAR, a Welch t-test was conducted to examine whether light perception provided any performance advantage during competition. A t-test was necessary because only two measures of VA were recorded for athletes with VA > 3.5 logMAR (i.e., 3.7 or 4.0 logMAR for those with or without light perception, respectively). The data were also examined by estimating a Bayes factor (comparing the fit of the data under the null hypothesis and the alternative hypothesis) because of the sample size relative to the number of variables.

#### Best Race Time

Results revealed no significant difference in the best race times of the athletes with light perception (*n* = 7; *M* = 0.74) and those without (*n* = 4; *M* = 1.11), *t*(8.97) = 0.80, *p* = 0.447, *d* = 0.47. Also, an estimated Bayes Factor suggested that the null hypothesis was 1.77 times more likely to be true than a model where there was a difference between the performance of those with and without light perception. This supports the preliminary suggestion that no further split is needed for athletes with VA > 3.5 logMAR.

#### Finish Time and Mean Lateral Position

Table 10 presents the main results for finish time and mean lateral position, that is for *t*-tests and Bayes Factors among athletes with VA > 3.5 logMAR. The conclusion related to finish time was the same as best race time, meaning that no further split was necessary when comparing athletes with and without light perception. However, with respect to mean lateral position, the Bayes Factor indicates that a further split could be made, with the alternate hypothesis 1.85 times more likely to be true than a model where there would be no difference between the performance of those with and without light perception.

**Table 10:** Results summary for finish time and mean lateral position for participants with VA > 3.5 logMAR

Performance measures	Main outcomes	
	*t* test	Bayes factor
Finish time	Light perception *n* = 7 *M* = 1.0 No light perception *n* = 4 *M* = 0.73 *t*(5.28) = 0.78, *p* = 0.47 *d* = 0.58	1.65 times more likely to have no effect of light perception on performance
Mean lateral position	Light perception *n* = 7 *M* = 0.62 No light perception *n* = 4 *M* = 0.82 *t*(6.73) = 2.32, *p* = 0.06 *d* = 1.6	1.85 times more likely to have an effect of light perception on performance

## Discussion

The aim of this study was to establish the relationship between visual function and performance in elite VI Para swimming. A battery of tests of visual function was administered to international-level VI swimmers whose performance results were obtained from international competitions. The results confirm the necessity of visual information for optimal swimming performance, with swimmers with better visual function outperforming those with only rudimentary or no vision. However, the relationship between visual function and performance was not linear. In particular, the results revealed no measurable difference in the overall swimming performance of those athletes who had measurable VA, irrespective of how good or bad their VA was. VA remained the visual function best able to predict the overall performance of the swimmers (i.e., when considering best race time). However, performance in specific aspects of the swim race were also related to some small degree to other visual function measures such as a swimmer’s depth perception, motion perception, light sensitivity and visual search ability. These results not only help to further our understanding of the impact of VI on swimming performance, but also suggest that modifications are necessary to the current classification system used in VI swimming in the Paralympic Games.

### Impact of VI on Swimming Performance

Despite criticism from experts that VA might not test an aspect of visual function vital for optimal swimming performance [7], the results of the present study show VA to be the *best* predictor of overall performance. Previous studies have indirectly inferred a relationship between VA and swimming performance by comparing the performance of the athletes in the existing sport classes [26–28], but our study goes beyond this to show that it remains the *best* predictor of performance even when including other tests that might be more representative of the visual demands inherent of swimming. The primacy of VA was evident not only when examining correlations between measures of visual function and performance, but also when performing a logistic regression which showed that VA alone best predicts the high and low performing athletes based on their best race time. This result is probably because VA, as a measure, is likely to be a good proxy for a variety of different tests of visual function. Many often question why the test of VA is used for classification given that the task, that is, to distinguish the direction in which an ‘E’ or a grating is pointing, is not representative of the visual demands of the sport. However, our results show that performance on the test of VA is highly correlated with numerous other measures of visual function which *are* assumed to be more functionally relevant in sport (e.g., DP, MP, CS; see Table 4). VA remains a good proxy for evaluating the overall capability of an athlete’s visual system.

Having established VA as the visual function most closely related to overall race time, the present study also looked at the potential influence of other visual functions on the performance of specific aspects of a swimming race. More specifically, the results revealed that depth perception, motion perception, light sensitivity, and visual search help to predict *high* and *low* performing swimmers based on their finish time or mean lateral position in the lane. However, the practical implications of those results appear minimal, with the percentage of correct classification in those models only marginally higher than that obtained when including VA alone, even with the addition of further predictors (i.e., less parsimonious models). In fact, a decrease in correct classification was even observed for the mean lateral position in the lane. Therefore, it remains questionable whether the benefits of including those additional measures of visual function to the classification procedure would outweigh the additional complexity and time associated with the inclusion of those measures. Nonetheless, it is noteworthy that the findings provide some support for the experts’ opinion regarding the importance of other aspects of visual function in understanding VI swimming performance [7].

### Implications for Classification in Para Swimming

Previous studies comparing performance across pre-existing vision classes in swimming (S13, S12, S11) have suggested that only two classes may be necessary for VI swimming [26–28]. However, those studies were not able to establish what should be the borders between the classes and what measures of visual function are best to delineate those classes. The present study addressed those shortcomings by directly measuring a range of visual functions to examine the continuous relationship between VI and swimming performance, rather than simply comparing the performance of different competition classes [5]. This approach allows us to make suggestions for empirically driven sports classes to improve classification for VI swimming.

The results of the present study are in agreement with the opinion of experts who had suggested that only two classes may be necessary to provide legitimate competition in VI swimming [7]. Indeed, our decision tree analyses support the idea that only two classes are necessary. A single split in performance was favoured at a VA of 3.5 logMAR for the best race time, and at 2.6 logMAR for the finish time and the mean lateral position in the lane. At first sight, the difference between these two values may seem substantial. However, in this and other studies, it is rare to find athletes with a binocular VA between 2.6 and 3.5 logMAR (which could be due to the way VA is generally measured [10, 16, 21, 23]). Indeed, there were no athletes with that level of acuity in our study. This suggests that a decision to place the split at either 2.6 logMAR or 3.5 logMAR is a relatively inconsequential one, except for the athletes with that level of acuity, because very few athletes have a VA within this range.

An important nuance to those results needs to be highlighted, which comes from the fact that bootstrapping the decision trees with replacement 10,000 times yielded a high variability between cut-offs, ranging mainly between 2.1 and 3.5 logMAR. Even more important, approximately 45% of the bootstrapped samples found no split at all in best race time according to VA (though a vast majority of trees found at least one split when considering finish time and mean lateral position in the lane). In other words, with a different sample of athletes, the decision tree could have found a different threshold, or no threshold at all (i.e., suggesting that all swimmers should compete together). Krabben and colleagues [21] also recently found a large range of VA cut-off points rather than a unique value when examining the relationship between VI and performance in judo. The authors explained that research might be able to provide, at best, a *range* of VA cut-offs, rather than a definitive single value. The options raised by Krabben and colleagues [21] on how to resolve the final cut-off include (1) setting the cut-off at a conceptual border between partially sighted athletes and functionally blind ones, (2) or at 2.9 logMAR, which is the highest numeric VA value measurable by the BRVT [35], (3) or that the decision should not be entirely scientifically based, but that it could also be an ethical issue that requires the input of multiple relevant stakeholders (e.g., athletes, coaches, scientists, sports philosophers).

The present study found little evidence to suggest that there would be any benefit of including a third class by separating the athletes who had some measurable visual function (i.e., VA < 3.5 logMAR) into multiple classes. Effectively, these results are in disagreement with the current system of classification whereby athletes in the S13 and S12 classes compete separately. The existing system implicitly suggests that S12 swimmers would have a disadvantage if they were to compete against S13 swimmers, however, our findings do not support this. Instead, S12 swimmers appear to have no disadvantage if competing against S13s. Given that some of our results showed relationships between specific visual functions (i.e., visual search, motion perception, light sensitivity and depth perception) and specific aspects of the race (i.e., finish time and mean lateral position in the lane), it would remain possible that a specific class of athletes may exist in the S12 and S13 classes who are disadvantaged because of an impairment to a visual function that is important for swimming, but is not yet assessed during classification. Therefore, one could ask whether competition would be more legitimate for those athletes if other visual functions would be included in the classification procedure. In other words, would there be a difference in the performance of athletes in classes newly formed on the basis of further measures of visual function? We found no relationship between any visual function and overall performance, finish time and mean lateral position in the lane for athletes with VA ≤ 3.5 logMAR (Fig. 3 and Table 9). It appears that there is little or no justification to split the S13 and S12 swimmers (or any other newly formed subgroups) by adding new measures of visual function to classification.

There was some suggestion that a split might be necessary within the group of functionally blind swimmers (i.e., with light perception or no light perception), though such a split is unlikely to ever be practically necessary. Athletes with light perception appeared to have a modest advantage in their ability to remain in the middle of the lane while swimming, even though they swim with blackened goggles during competition. Caution is warranted though given the low participant numbers within that group (i.e., seven swimmers with light perception and four without) and that the association was only with the mean lateral position in the lane and not with overall race time or any other measures of performance. Moreover, given the relatively low number of athletes with only light perception or no light perception taking part in VI competition [10, 16, 21, 23], a split within that class would result in two classes with very few athletes and therefore in a relatively low level of competition.

Within our study, the performance of the S11 swimmers was evaluated while swimming in competition with blackened goggles. It remains possible that the S11 athletes with light perception *could* have performed better if allowed to swim without the goggles. If that would have been the case, then it would have provided further support for the need to consider splitting those S11 swimmers into two separate classes. However, this would only apply if a decision was made to allow those swimmers to compete without blackened goggles (or even for them to compete against those athletes with measurable visual function). However, in our experience, those athletes with light perception anecdotally report that their visual function is so rudimentary that the benefit of swimming without goggles is negligible. Moreover, the experts in VI swimming remain largely satisfied with the use of blackened goggles during competition and so there is no plan to change the current requirement for S11 athletes to wear blackened goggles [7].

An important principle in evidence-based classification is that classification should seek to place athletes into classes based on a loss of function resulting from their impairment, and not based on a skill that will improve as a result of training [1]. This vital principle guided the way both vision impairment *and* performance were measured in the present study. The measures of vision impairment were chosen to represent fundamental characteristics of visual function (e.g., visual acuity, contrast sensitivity, motion perception) that should not improve as a result of sport-specific training [6]. That choice makes those measures suitable for use during classification if they limit sperformance. We then sought to establish the impairment–performance relationship by relating sport isual function to determinants of swim performance (e.g., race time, turn time, position in lane) in a group of VI swimmers. Those determinants are indeed likely to improve as a result of training, which is why we statistically controlled for each athlete’s estimated training volume when quantifying the impairment–performance relationship. This helped us to achieve our goal of establishing the degree to which each visual function may limit an athlete’s ability to perform those skills that represent vital determinants of swimming performance.

Finally, this study is limited by the fact that only 45 100 m freestyle swimmers were included. Although this represents a considerable proportion of the international VI swimming population, it does limit the strength of the conclusions that can be made using some of the more advanced statistical analyses we have employed, in particular when using the decision trees and the bootstrapping to make a split given the low numbers of participant with VA ≥ 3.5 logMAR.

## Conclusions

The present study sought to further the development of a sport-specific system of classification in VI swimming by examining the relationship between visual function and performance in the sport [5, 32]. Our findings suggest that a two-class system with a separation based on VA (and only VA) somewhere between 2.6 and 3.5 logMAR would provide legitimate competition for athletes with VI. The opinions of key stakeholders in the sport and Paralympic movement (e.g., athletes, coaches, scientists, sport philosophers) would be useful to establish the most suitable cut-off between the two classes.

## Data Availability

The datasets generated during and/or analysed during the current study are available from the corresponding author on reasonable request.

## Footnotes

1 Two measures of visual function are used to classify athletes: (1) visual acuity, a measure of the sharpness of central vision, and (2) visual field, a measure of the size of the area which is seen. An athlete is allocated class B3 when they have a VA between 1.0 and 1.4 logMAR inclusive, or if their visual field is less than 40 degrees diameter. Athletes are allocated to the B2 class if their VA is between 1.5 and 2.6 logMAR inclusive, or their visual field is less than 10 degrees diameter. Finally, B1 can only be allocated based on VA, which must be greater than 2.6 logMAR [7].

2 We also ran the decision tree by entering all the visual functions as predictors and found the same result, suggesting that VA shares too much variance with other visual functions for them to find their own split in the data.

3 Note that the interpretation of these results requires caution because of the low participant numbers. A suitable sample size for this analysis would typically have been *N* ≈ 150 (*N* = 10 *k*/*p*, where *p* is the smallest of the proportions of negative or positive cases and *k* is the number of predictors, here (10*6)/0.41; [41]). Nonetheless, no other predictors were close to reaching significance.

## Code availability

The code generated during the current study is available from the corresponding author on reasonable request.
